# First report and evidence of multiple origins of diflubenzuron resistance alleles in *Culex pipiens* mosquito from Cyprus

**DOI:** 10.1186/s13071-025-06889-7

**Published:** 2025-06-20

**Authors:** Valentina Mastrantonio, Marlen Vasquez, Gregoris Notarides, Eleni Patsoula, Valentina Lucchesi, Flavio Piras, Romeo Bellini, Daniele Porretta

**Affiliations:** 1 Department of Environmental Biology, Sapienza University of Rome, Rome, Italy; 2 Department of Chemical Engineering, Cyprus University of Technology, Limassol, Cyprus; 3 Department of Public Health Policy, School of Public Health, University of West Attica, Athens, Greece; 4 Medical and Veterinary Entomology Department, Centro Agricoltura Ambiente “G. Nicoli”, 40014 Bologna, Italy

**Keywords:** Insecticide resistance, Vector control, Insect growth regulators, Diflubenzuron (DFB), chitin-synthase, Mosquitoes

## Abstract

**Background:**

Insecticide resistance is one of the primary problems affecting vector control worldwide. Assessing the occurrence of resistant alleles and understanding their origin across the geographic range of vector species is crucial for effective resistance management. In populations of the mosquito *Culex pipiens*, point mutations conferring resistance to the insecticide diflubenzuron (DFB) were recently found across the Mediterranean basin. In this study, we investigated the possible occurrence of DFB resistance in Cyprus, where West Nile virus outbreaks have been documented in recent years.

**Methods:**

We sequenced a fragment of the chitin-synthase 1 gene carrying the resistant mutations in individuals collected from 18 populations of *Cx. pipiens* in Cyprus to investigate the occurrence of DFB-resistant alleles. We then assessed the evolutionary origin of DFB-resistant alleles by reconstructing the phylogenetic relationships between susceptible and resistant alleles found across the Mediterranean basin.

**Results:**

Our screening revealed the occurrence of the I1043F allele in all the districts analyzed. Notably, a new gene codon underlying the I1043F allele was detected. To our knowledge, this has not been previously reported in areas with DFB-resistance alleles in *Cx. pipiens*. In addition, we observed that the I1043F alleles detected in Cyprus have a different genetic background from those reported in other geographic areas, such as Italy and Turkey.

**Conclusions:**

To our knowledge, this is the first time in which DFB resistance was revealed in *Cx. pipiens* populations occurring in Cyprus. Furthermore, we demonstrate that I1043F-resistant alleles have an independent origin in Cyprus, further supporting the hypothesis of a multiple independent origin of DFB resistance across the Mediterranean region. These results stress the need for regular resistance surveillance activities and the urgency of developing new mosquito control strategies.

**Graphical abstract:**

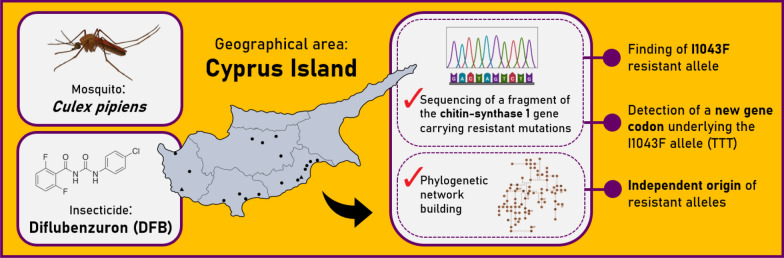

**Supplementary Information:**

The online version contains supplementary material available at 10.1186/s13071-025-06889-7.

## Background

Vector-borne diseases, accounting for more than 700,000 deaths annually, represent a major concern in public health [1]. Mosquitoes, the primary vectors of human diseases such as malaria, dengue, chikungunya, and West Nile fever, require specific control efforts since no effective vaccines are available. Chemical insecticides remain a cornerstone in mosquito control. However, the evolution of insecticide resistance strongly compromises this method, as resistant populations are recurrently reported in the major mosquito vectors, such as *Anopheles*, *Aedes*, and *Culex* [2–4].

*Culex pipiens* is the primary vector of West Nile virus (WNV) in Europe [5]. Diflubenzuron (DFB), an insect growth regulator acting as a chitin synthesis inhibitor, has been largely employed against this species [6]. Indeed, DFB-based applications have been regularly implemented in vector control programs of several countries within the geographic range of *Cx. pipiens*, such as Italy, Turkey, and Greece [7–9]. DFB interacts with the chitin synthase 1 gene (*chs1*), causing insect death and abortive molting [10, 11]. Concerns about the effectiveness of DFB in mosquito control have emerged in recent years because some individuals of *Cx. pipiens* were found to be resistant in Italian populations [7, 12–14]. Genetic analyses showed that DFB resistance is associated with point mutations in position 1043 of the *chs1* gene. While susceptible individuals have a nucleotide sequence coding for isoleucine (ATC), resistant individuals have a sequence coding for leucine (ATC→CTC, I1043L, resistance ratio > 20-fold), methionine (ATC→ATG, I1043M, resistance ratio > 15,000-fold), and phenylalanine (ATC→TTC, I1043F, resistance ratio > 15,000-fold) [12, 14]. Since the first detection in the Italian population of *Cx. pipiens*, DFB mutations have been found in many other regions across the Mediterranean basin, including France, Turkey, and Crete [8, 9, 12], and phylogenetic data revealed that they emerged multiple independent times across *Cx. pipiens*’ geographic distribution [15]. These findings are alarming and highlight the urgent need for systematic DFB resistance monitoring throughout the geographic range of *Cx. pipiens*. Indeed, DFB is an indispensable tool for *Cx. pipiens* control since it remains one of the limited larvicides that is still effective against mosquitoes. Therefore, understanding the occurrence of DFB-resistant mutations is crucial for preserving its efficacy and planning successful control strategies, especially in areas where WNV outbreaks caused by *Cx. pipiens* have been documented in recent years [16, 17].

In this paper, we investigated the occurrence of DFB-resistant alleles in Cyprus. While *Cx. pipiens* is widespread and was responsible for the 2019 WNV outbreak [17], as well as for the yearly recorded WNV cases, data regarding DFB resistance in *Cx. pipiens* populations from Cyprus are currently not available. This is particularly relevant given that DFB is among the chemicals primarily used in urban areas throughout the island against insect pests [18, 19].

## Methods

**Figure 1:**
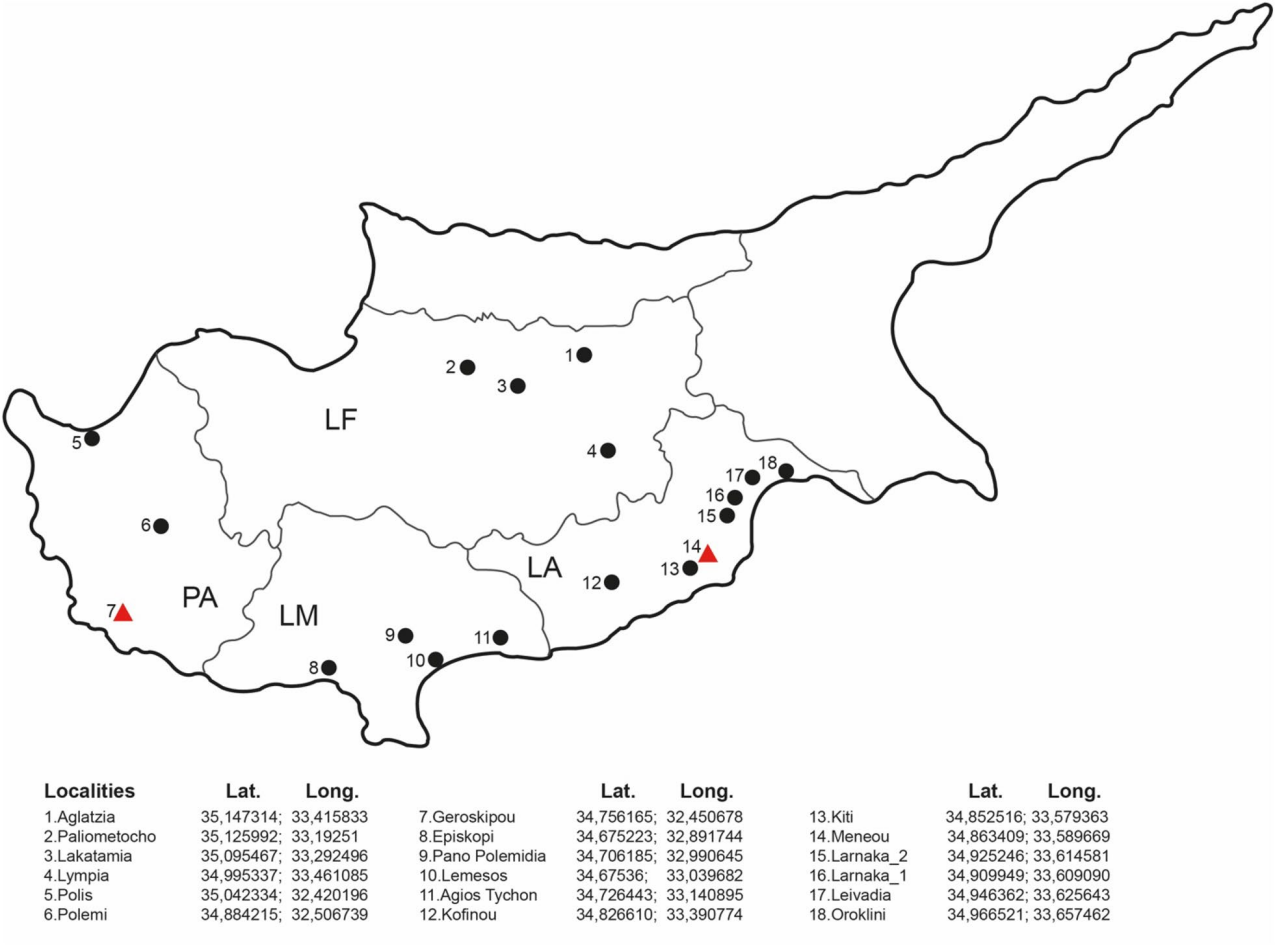
Map showing the sampling sites of *Culex pipiens* across four districts of Cyprus: LF, Lefkosia district; PA, Pafos district; LM, Lemesos district; LA, Larnaka district. Red triangles are localities sampled in 2022; black dots are localities sampled in 2023

We collected *Cx. pipiens* adults from 18 localities spanning four districts across Cyprus from November 2022 to July 2023 within a national surveillance under the IAEA CYP5020 project (Fig. 1). The sampling strategy was planned according to previous sampling campaigns focusing on the detection of WNV in mosquitoes in “high-risk” areas. High-risk areas are areas in which at least one of the following criteria are met: (1) previous WNV human cases reported; (2) previous WNV detection in birds or horses; (3) highly populated urban or semiurban areas; and (4) known presence of *Cx. pipiens* at high numbers or invasive *Aedes* species. Proximity to main roads and the safety of equipment were also taken into consideration in the sampling design. BG-Sentinel traps (Biogents, Germany) baited with 1 kg of dry ice were used. Traps were set every 2 weeks overnight for less than 24 h and collected between 9:20 and 15:30. To increase the detection capability and achieve better geographic coverage, only catches of > 7 *Cx. pipiens* from as many different locations as possible were used. Each pool consisted of 7–9 adults. The catch was transferred to the laboratory and morphologically identified using a cold table and the keys [20, 21]. *Cx. pipiens* female and male mosquitoes for each sampling date were pooled into 2 ml Eppendorf tubes and stored at −80 °C until further analysis. For this study, a selection of pools was made to have at least one pool from the sites monitored and from as many dates as possible to test different *Cx. pipiens* mosquito populations.

Genomic DNA was extracted from single mosquitoes using the C-TAB method [22]. After DNA extraction, a fragment of the *chs1* gene including the 1043 codon was amplified by polymerase chain reaction (PCR) using the primer pair CHSseqF (5′-CCGCGTTCAAGATTGACAACTGG-3′) and CHSseqR (5′-TCCAGTAGGGGTTCGTCAGG-3′) and the protocol described in Grigoraki et al.  [14]. PCR products from each individual were then double-stranded sequenced by standard Sanger sequencing by Microsynth Inc., Balgach, Switzerland [23].

The sequences were checked and aligned using the software Chromas 2.6.5 (Technelysium, Helensvale, Australia) and Clustal X 2.149 [24], respectively. Heterozygous positions were identified by double peaks in the electropherograms [25] and were encoded using the IUPAC ambiguity code. The phase algorithm [26], implemented in DnaSP v.6 with default settings was used to resolve genotypes of heterozygous individuals. Between the *chs*-allele pairs reconstructed, we considered exclusively those with Bayesian posterior probabilities (BBP) of value > 90% [27]. All unique sequences were submitted to GenBank (accession codes: PQ898938-PQ898997). Diversity indices (i.e., polymorphisms of nucleotide sequences, gene, and nucleotide diversity) and the average uncorrected *p*-distance between *chs*-alleles were also calculated using the software DNASP v. 6.0. [28] and MEGA 7.051 [29], respectively. Genealogical relationships among *chs*-alleles were inferred by constructing a phylogenetic network using the statistical parsimony algorithm with a 95% cutoff implemented in the TCS software [30, 31]. The *chs*-alleles found in our previous studies on DFB resistance in *Cx. pipiens* populations from Italy and Turkey (accession codes: MZ494156-MZ494187) were also included in the phylogenetic analysis [15].

## Results and discussion

We obtained sequences of 608 bp of the *chs-1* gene encompassing the site 1043 of 201 *Cx. pipiens* individuals collected across Cyprus. Our screening for DFB-resistant alleles revealed the occurrence of the I1043F allele in all the districts analyzed (Table 1; Supplementary Table 1), while no evidence was obtained on the other alleles known to confer DFB resistance. Notably, we found two gene codons underlying the I1043F allele: the TTC codon, previously found in other *Cx. pipiens* populations, and the TTT codon that, to our knowledge, has never been previously found (Table 1). The I1043F resistant allele was found in 7 out of 18 localities and in both years of sampling, with the highest I1043F frequency detected in the Larnaka localities (up to 0.55) (Supplementary Table 1). Interestingly, diflubenzuron is used regularly in Larnaka city to control mosquito populations within the storm water distribution system (M.V. personal observation).

**Table 1:** Allelic frequency of wild-type and DFB-resistant alleles in *Culex pipiens* populations from Cyprus

District	*N*	Allelic frequencies				
		I	L	M	*F*	
		(ACT)	(CTC)	(ATG)	(TTC)	(TTT)
Lefkosia	46	0.95	–	–	–	0.05
Pafos	36	0.97	–	–	0.03	–
Lemesos	48	0.94	–	–	0.06	–
Larnaka	65	0.84	–	–	0.09	0.07

N, number of individuals analyzed in each district; I, wild-type susceptible allele I1043; L, resistant allele I1043L; M, resistant allele I1043M; F, resistant allele I1043F. The gene codon underlying each mutation is also shown.

**Figure 2:**
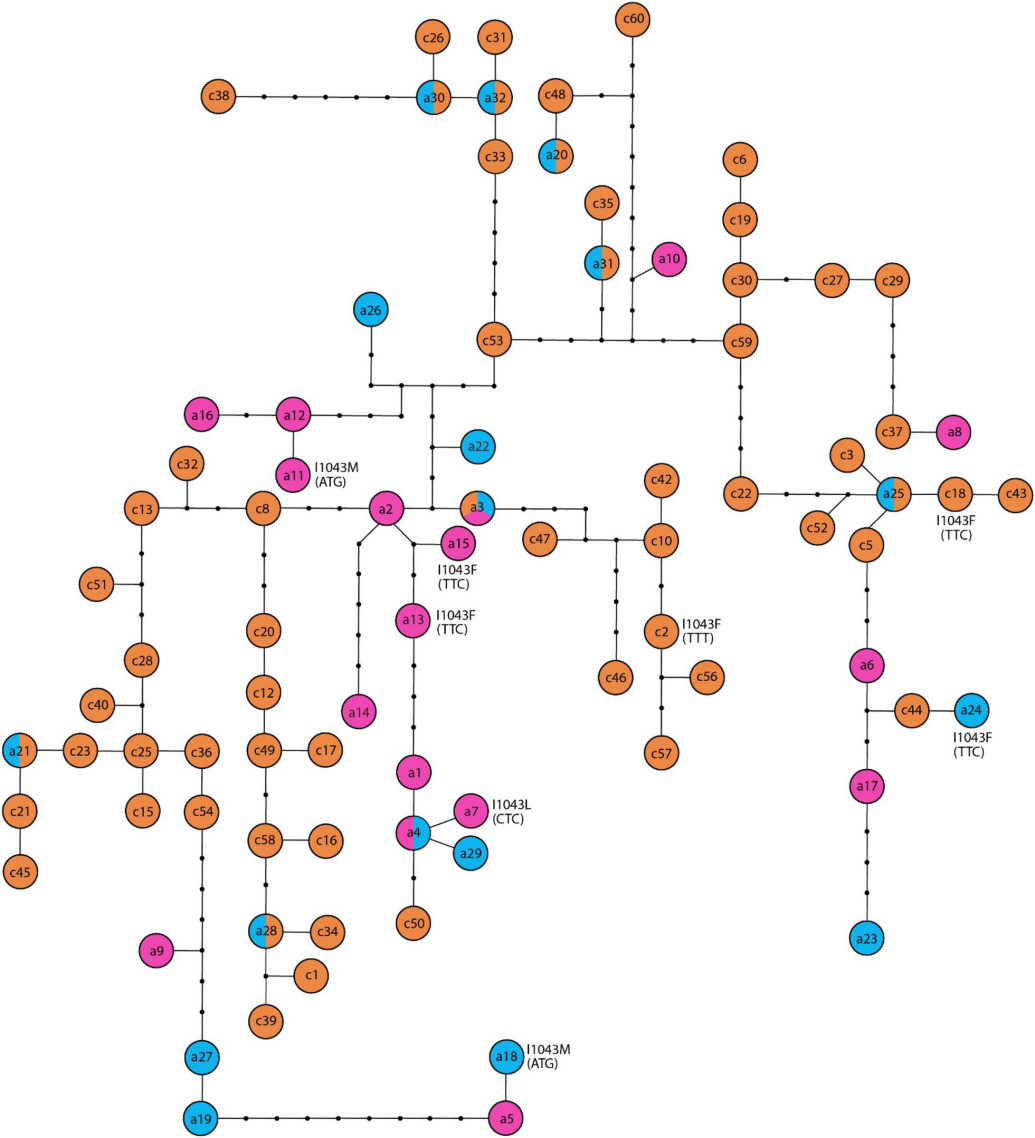
Phylogenetic network among the *chs*-alleles of *Cx. pipiens*. The network was constructed using unique *chs-1* sequences from Cyprus (orange), Italy (bright pink), and Turkey (light blue). For resistant alleles, the sequence at the 1043 codon is also shown (shared alleles: a3 = c14; a20 = c55; a21 = c24; a25 = c7; a28 = c41; a30 = c9; a31 = c11; a32 = c4). Circle represents single alleles; dots mean missing alleles. The frequency of individual haplotypes in each locality is provided in Supplementary Table 1

Among the 201 individuals analyzed, 178 were heterozygous at more than one site, and 33 were homozygous at all nucleotide positions. Phased allele sequences showing BBP > 90% were obtained for 115 individuals. DNA polymorphism analysis revealed the occurrence of 60 *chs*-alleles (encoded as c1-c60) (Supplementary Table 1) identified by 40 polymorphic sites, 37 of which were parsimony informative. The mean gene diversity and nucleotide diversity between the *chs*-alleles were 0.937 (SD 0.007) and 0.0163 (SD 0.0003), respectively. The average uncorrected *p*-distance was 0.017 (SE 0.003). Phylogenetic analysis supported a scenario of multiple independent origins of the DFB-resistant alleles. Indeed, the two codons encoding for the phenylalanine were found in two different *chs*-alleles separated by multiple nucleotide substitutions (Fig. 2). Interestingly, we also observed that the I1043F alleles detected in Cyprus have a substantially different genetic background from those found in Italy and Turkey (Fig. 2), which again suggests an independent origin of DFB resistance in Cyprus.

Our findings expand the knowledge of the geographic distribution of DFB resistance and add further evidence to the multiple evolutionary origins of DFB-resistant alleles across the Mediterranean region. Furthermore, they call for significant public health interest and concern, as DFB is applied in most European countries for mosquito control, including *Cx. pipiens* and *Aedes* mosquito vectors. DFB is among the few active substances approved by European legislation and available in the continent (EU Biocides Regulation 528/2012) for vector control [18]. The occurrence of the DFB-resistant I1043F allele in Cyprus is worthy of attention, as DFB resistance, combined with the reduced availability of reliable biocides for mosquito control [32], highlights the need to develop appropriate insecticide resistance management (IRM) strategies and/or implement new mosquito control methods. These may include applying alternative vector control strategies and methodologies to ensure effective suppression of these species. Outbreaks of vector-borne diseases and invasive mosquito species are indeed expected to rise in the near future. For this reason, it would be appropriate to investigate whether *chs-1* resistance mutations are present in other relevant mosquito vectors in Cyprus, such as *Aedes aegypti* and *Ae. albopictus*.

## Supplementary Information

Additional file 1.

## Data Availability

The manuscript and supplementary files provide all data supporting the main conclusions. All sequences generated in the study were deposited in GenBank, and the accession numbers are provided in the Methods section.
