# Supplementary material for: First report and evidence of multiple origins of diflubenzuron resistance alleles in Culex pipiens mosquito from Cyprus
Source: Parasit Vectors. 2025 Jun 20;18:231. doi: 10.1186/s13071-025-06889-7 (PMC12180259; doi:10.1186/s13071-025-06889-7)
Supplement: Supplementary file 1 — Additional file 1. [file 13071_2025_6889_MOESM1_ESM.docx]

Parasites & Vectors

Brief Report

**First report and evidence of multiple origins of diflubenzuron resistance alleles in *Culex pipiens* mosquito from Cyprus**

Valentina Mastrantonio^1*^, Marlen Vasquez^2^; Gregoris Notarides^2^; Eleni Patsoula^3^; Valentina Lucchesi^1^, Flavio Piras^1^, Romeo Bellini^4^, Daniele Porretta^1^

| **Code** | **Locality** | **District** |  | **N** | **Genotypic frequency** | | | | | | | | | | **chs-alleles** |
| --- | --- | --- | --- | --- | --- | --- | --- | --- | --- | --- | --- | --- | --- | --- | --- |
|  |  |  |  |  | **II** | **IL** | **LL** | **IM** | **MM** | **LM** | **FF** | **IF** | **FL** | **FM** |  |
| 1. | Aglatzia | Lefkosia |  | 7 | 6 | - | - | - | - | - | - | 1 | - | - | c1, **c2** |
| 2. | Paliometocho | Lefkosia |  | 11 | 11 | - | - | - | - | - | - | - | - | - | c3(3), c4(3), c5, c6, c7(3), c8, c9(2), c10, c11, c12, c13 |
| 3. | Lakatamia | Lefkosia |  | 12 | 10 | - | - | - | - | - | - | 2 | - | - | c4, c7, c9, c11, c14, c15(2), c16, c17, **c18**, c19, c20 |
| 4. | Lympia | Lefkosia |  | 11 | 10 | - | - | - | - | - | - | 1 | - | - | c11(2), c14(5), **c18**, c21, c22 |
| 5. | Polis | Pafos |  | 12 | 12 | - | - | - | - | - | - | - | - | - | c9(2), c11(3), c14, c23, c24 |
| 6. | Polemi | Pafos |  | 12 | 10 | - | - | - | - | - | - | 2 | - | - | c4, c7(3), c11(4), c15(2), **c18**(2), c19(2), c25(2), c26, c27, c28, c29 |
| 7. | Geroskipou | Pafos |  | 12 | 12 | - | - | - | - | - | - | - | - | - | c3, c4(2), c7(3), c9(2), c11(2), c14, c25, c27, c30(2), c31 |
| 8. | Episkopi | Lemesos |  | 12 | 12 | - | - | - | - | - | - | - | - | - | c3, c4(2), c7, c9(2), c11(5), c14, c15(2), c27, c32, c33, c34(2), c35, c36, c37 |
| 9. | Pano Polemidia | Lemesos |  | 12 | 12 | - | - | - | - | - | - | - | - | - | c4(4), c7(7), c8, c10, c11, c12, c19 |
| 10. | Lemesos | Lemesos |  | 12 | 12 | - | - | - | - | - | - | - | - | - | c7(8), c9(3), c11(2), c15, c19, c38, c39, c40 |
| 11. | Agios Tychon | Lemesos |  | 12 | 7 | - | - | - | - | - | 1 | 4 | - | - | c4(6), c7(2), c11(2), **c18**(4), c41, c42, c43, c44 |
| 12. | Kofinou | Larnaka |  | 11 | 11 | - | - | - | - | - | - | - | - | - | c4, c14, c16, c41(3), c45, c46, c47, c48 |
| 13. | Kiti | Larnaka |  | 11 | 11 | - | - | - | - | - | - | - | - | - | c4(3), c7(3), c11, c12, c27, c30, c40, c44, c49, c50 |
| 14. | Meneou | Larnaka |  | 11 | 10 | - | - | - | - | - | - | 1 | - | - | c51, c52, c53, c54, c55(2) |
| 15. | Larnaka_2 | Larnaka |  | 11 | 4 | - | - | - | - | - | 4 | 3 | - | - | **c2**(3), c7, c14(2), **c18**(5), c21, c56, c57 |
| 16. | Larnaka_1 | Larnaka |  | 11 | 3 | - | - | - | - | - | 4 | 4 | - | - | **c2**(4), **c18**(5), c19 |
| 17. | Leivadia | Larnaka |  | 10 | 10 | - | - | - | - | - | - | - | - | - | c7(3), c11, c14(5), c15, c24, c58 |
| 18. | Oroklini | Larnaka |  | 11 | 11 | - | - | - | - | - | - | - | - | - | c7, c9, c59, c60 |

**Supplementary Table 1**. **Genotypic and allelic frequency of DFB resistance alleles in *Culex* *pipiens* populations from Cyprus**. N= number of individuals analysed in each locality. I = wild-type susceptible allele I1043; L = resistant allele I1043L; M = resistant allele I1043M; F = resistant allele I1043F. The DFB-resistant alleles are shown in bold.
